# Increase in white blood cell counts by pegbovigrastim in primiparous and multiparous grazing dairy cows and the interaction with prepartum body condition score and non-esterified fatty acids concentration

**DOI:** 10.1371/journal.pone.0245149

**Published:** 2021-01-07

**Authors:** Joaquín Barca, Ynte H. Schukken, Ana Meikle

**Affiliations:** 1 Department of Dairy Science and Technology, Veterinary Faculty, Montevideo, Uruguay; 2 Department of Animal Sciences, Wageningen University, Wageningen, The Netherlands; 3 GD Animal Health, Deventer, The Netherlands; 4 Animal Endocrine and Metabolism Laboratory, Veterinary Faculty, Montevideo, Uruguay; Michigan State University, UNITED STATES

## Abstract

The objective of this study was to determine if parity affected the effect of pegbovigrastim (PEG) treatment on white blood cell (WBC) counts in grazing dairy cows. Additionally, the association of prepartum body condition score (BCS) and non-esterified fatty acid (Pre-NEFA) concentration with WBC counts was investigated. The effect of early-lactation disease was included in the statistical analysis. A randomized controlled trial on four commercial grazing dairy farms was performed. Holstein primiparous (Control = 87, PEG = 89) and multiparous (Control = 181, PEG = 184) cows were randomly assigned to one of two treatments: first PEG dose 8 ± 5 (mean ± SD) days before the expected calving date and a second dose within 24 h after calving (PEG) compared to untreated controls (Control). Treatment effects were evaluated with mixed linear regression models. Treatment with PEG increased WBC, neutrophil, lymphocyte and monocyte counts at 6 ± 1 (mean ± SD) days in milk. Parity, BCS and their interactions with treatment were not associated with WBC counts. In control cows, Pre-NEFA concentration was associated with reduced WBC, neutrophil and lymphocyte counts and tended to be associated with reduced monocyte counts. Pegbovigrastim treatment reversed the negative association of Pre-NEFA concentration with neutrophil and monocyte counts and tended to reverse the negative association of Pre-NEFA concentration with WBC counts. In the PEG treated group, cows diagnosed with retained placenta or metritis showed lower neutrophil counts when compared to PEG treated cows without these clinical diseases. These data confirm that PEG treatment increases WBC, neutrophil, lymphocyte and monocyte counts in grazing dairy cows and that this effect is independent of parity. Pegbovigrastim treatment reversed the negative association of Pre-NEFA concentration with neutrophil and monocyte counts, and tended to reverse the negative association of Pre-NEFA concentration with WBC counts.

## Introduction

Around 50% of dairy cows experience a metabolic or infectious disease or both during the first month of lactation. The risk for disease in early lactation has been associated with, among other causes, the negative energy balance (NEB) that takes place during the transition period [1]. Metabolites related to NEB such as non-esterified fatty acids (NEFA) and beta-hydroxybutyrate (BHB), have been linked to immunosuppression and increased risk of infectious and clinical diseases [2–4]. In early lactation, when NEB it is typically most profound (i.e. higher NEFA concentration), several studies have shown a decline in the neutrophil and lymphocyte counts as well as impaired function (i.e. reduced chemotaxis, phagocytosis and oxidative burst, and reduced proliferative capacity, respectively) [4–6]. Although there are reports on the negative effect of NEB on neutrophil and lymphocyte function [2, 6, 7], there are few reports on the effect of NEB on the counts of these immune cells. It has been shown that cows with increased postpartum NEFA concentrations (> 0.5 mM) had decreased white blood cell (WBC) counts [8]. Conversely, in a controlled trial [9], no significant effect of prepartum energy restriction on WBC count was found relative to non-restricted control cows. However, in this trial [9], both groups presented a high frequency of cows with high (defined as > 0.4 m*M*) NEFA concentrations one week before calving (56% vs 85% non-restricted vs restricted, respectively). In vitro, it has been shown that NEFA concentrations markedly decreased the viability of neutrophils [10]. Over-conditioned cows mobilize more fat during the transition period [11] and this fat mobilization is associated with higher NEFA concentrations [12]. It has been suggested that under-conditioned cows tended to have decreased neutrophil counts [13].

Parity is an important factor determining health and metabolic events during transition to lactation. Compared with multiparous cows, primiparous cows before their first calving had a less pronounced NEB (in terms of NEFA concentration) in confined housing [14]. However in pasture-based herds it has been reported that primiparous cows have a more pronounced NEB compared to multiparous animals [15, 16]. Parity also modifies the risk for disease: Reinhardt et al. [17], in a large number of US farms, reported that primiparous cows had a lower risk for hypocalcemia. Toni et al. [18] reported that housed primiparous cows had a higher risk for metritis. In both confined and grazing primiparous cows, clinical mastitis (CM) incidence is typically higher during early lactation, particularly in the first week postpartum, while for the whole lactation it is lower than in multiparous cows [19]. Interestingly, an early study reported that older cows had impaired neutrophil function [20]. In older cows, a decrease in lymphocyte types (γδT cell and B cells) has been shown, which might be associated with increased susceptibility to infection. [21].

The use of tools to reduce disease incidence at the start of the lactation is of great interest. The treatment with a polyethylene glycolated form of recombinant bovine G-CSF (PEG, or Pegbovigrastim, marketed as Imrestor®, Elanco Animal Health, Greenfield, IN) in periparturient dairy cows has been reported to be beneficial, as treatment increased the number of circulating WBC, neutrophil, lymphocyte and monocyte counts [9, 22, 23]. It is currently not known whether parity affects the impact of PEG on WBC counts. Using gene expression data, it was suggested that PEG treatment improved migration, adhesion, and antimicrobial capacity and enhanced the inflammatory response regardless of parity [24].

The response to PEG treatment maybe modulated by the metabolic status of the cow since metabolic markers (NEFA) affect the immune system. However, in a recent study, prepartum energy restriction did not affect the WBC count in response to PEG in comparison with controls [9].

Thus, we hypothesized that parity affects the response to PEG as measured by WBC counts in grazing dairy cows, and that the response to treatment may be associated with prepartum BCS and NEFA (Pre-NEFA) concentration. Therefore, we investigated the effect of PEG treatment on postpartum (5 to 8 DIM) WBC counts in primiparous and multiparous grazing dairy cows. Additionally, the association of BCS and Pre-NEFA concentration with WBC counts was investigated. The effect of early-lactation disease was included in the statistical analysis.

## Materials and methods

The experimental protocol (CEUAFVET-PI-162) was evaluated and approved by the Honorary Committee for Animal Experimentation in Uruguay (CHEA), University of Uruguay.

### Experimental design

A subset of Holstein pregnant heifers (Primiparous, n = 194) and cows that were approaching their second or higher calving (Multiparous, n = 399) from a larger prospective controlled randomized trial was enrolled in this experiment. The trial was performed on 4 commercial grazing dairy farms in 3 different regions of Uruguay (San José, Florida, Rio Negro). Primiparous and multiparous cows on each farm were located in outdoor close-up paddocks around 3 weeks before the expected calving date (ECD) and remained in this paddock until calving. Both groups were followed for approximately 1 week postpartum (5 to 8 DIM), at which point blood sampling for WBC counts was performed.

Cows from farms 2, 3, and 4, were managed in separate groups based on parity, but under the same environmental conditions, including milking and feeding management. Farm 1 managed a single group after calving. Calving occurred from February 21^st^ to July 24^th^ of 2018. After calving, animals were kept on pasture and at least 40% of the dry matter intake (DMI) came directly from grazing. The diet was supplemented with a partial mixed ration. Exceptionally, when weather conditions did not allow grazing, cows were kept in outdoor paddocks. All cows were milked twice a day.

**Fig 1:**
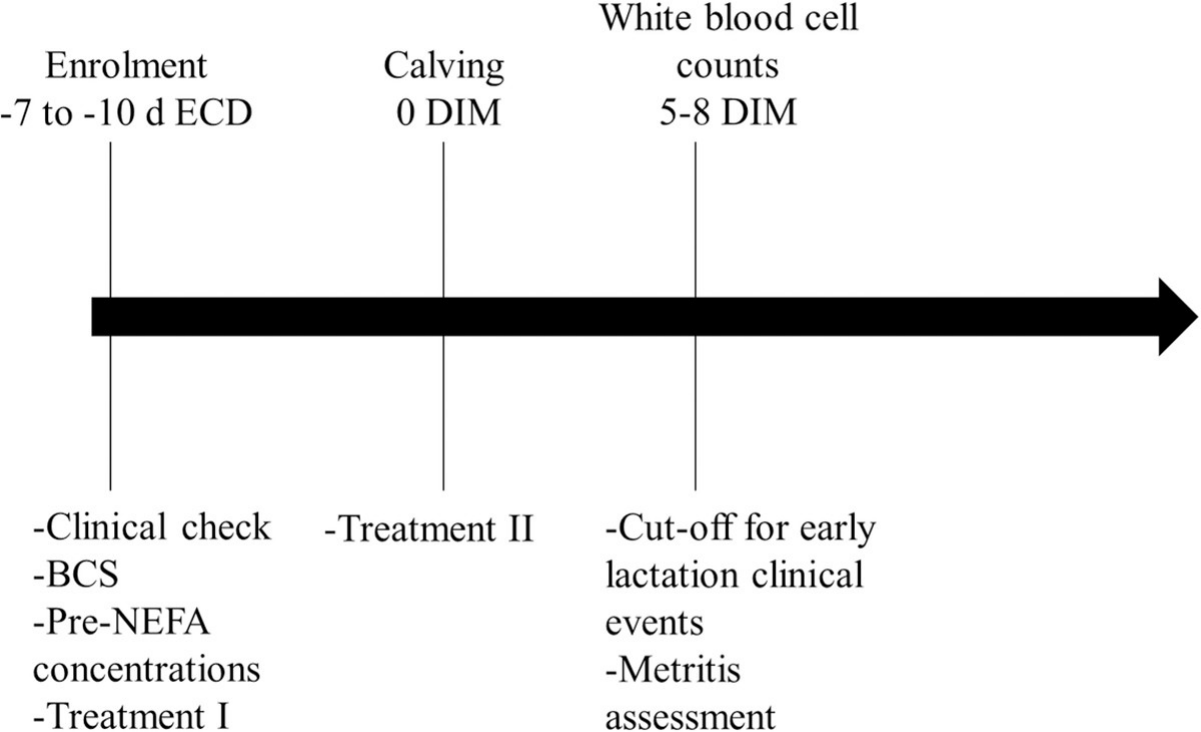
Diagram with time points and measurements taken. ECD: expected calving date, Pre-NEFA: prepartum NEFA concentration.

Fig 1 provides a diagram with the relevant time points and event measurements in the present study. Primiparous and multiparous cows in the close-up paddocks were assessed twice weekly. Cows that were between -10 to -7 days relative to the ECD were clinically examined to assess whether any of the exclusion criteria, i.e. clinical disease and/or fever (rectal temperature > 39.5°C) were present. When none of the exclusion criteria were met, BCS was assessed [25], blood samples were collected for NEFA determination and animals were assigned to one of two treatments. Cows with even national ear tag number were injected with PEG (Imrestor®, Elanco Animal Health, Greenfield, IN) according to the product label (PEG) and animals with an odd national ear tag number remained as untreated controls (Control). Animals assigned to PEG treatment received a second dose within 24 h after calving. Only cows that received both doses were included in the study. The included animals therefore represent the ‘per protocol’ inclusion rule.

### Clinical diagnoses and definitions

Clinical mastitis was diagnosed by trained farm personnel during forestripping at each milking and defined according to Pinzón-Sánchez and Ruegg [26]. Retained placenta (RP) was defined according to Ruiz et al. [27]. Puerperal metritis and clinical metritis were defined according to Sheldon et al. [28]. Fever was defined as a rectal temperature >39.5°C and >40.5°C during summer and when ambient temperature was higher than 30°C [29]. In the present study, all CM cases, irrespective of severity, were defined as CM. Puerperal metritis and clinical metritis as defined according to Sheldon et al. [28] were grouped and reported as metritis.

At the postpartum visit, at 5 to 8 DIM, all cows were carefully assessed to diagnose metritis. If metritis was diagnosed by the study personnel before this time point, it was also recorded and included in this disease category.

### Non-esterified fatty acid and white blood cell count determination

Blood samples were collected from the coccygeal vessels (8.5-mL clot accelerator tubes, Becton Dickson, Franklin Lakes, NJ). Immediately, samples were centrifuged at 3000 x g for 20 min and serum was stored frozen (-20°C) until further analysis. Serum was analyzed for NEFA concentration at the Animal Endocrine and Metabolism laboratory, Veterinary Faculty, Montevideo, Uruguay. Non-esterified fatty acid (NEFA) concentrations were measured by colorimetric assays on an A25 autoanalyzer (© Biosystems S.A., Barcelona, Spain) using commercial kits: Wako NEFA-HR (2), Wako Pure Chemical Industries Ltd., Osaka, Japan. The inter-assay coefficient of variation (CV) for commercial quality controls was less than 10%.

At approximately 1 week postpartum (5 to 8 DIM), a second blood sample was taken in K2- EDTA 6.5-mL tubes (Becton Dickson, Franklin Lakes, NJ) from the coccygeal vessels and sent immediately to the laboratory for total and differential WBC count. The total WBC count was determined using an automated haemocytometer (Sysmex XT1000, Roche Diagnostic, CA, USA) and cell morphology was assessed by microscopic examination of blood smears. The inter-assay CV for commercial quality controls was less than 5%.

### Statistical analysis

Data were analyzed using the SAS software (SAS Institute Inc. 2018. SAS® University Edition, Cary, North Carolina: SAS Institute Inc.). Descriptive statistics were performed using the t-test procedure (PROC TTEST) and chi-squared test (PROC FREQ) for continuous and discrete variables (i.e. occurrence of CM cases) respectively.

As the occurrence of inflammatory clinical diseases such as CM, RP, and metritis close to the time of measurement of WBC counts would likely impact these counts [30], we considered the occurrence of these diseases in the analysis of the WBC data. Clinical mastitis, RP, and metritis, occurring up until the time of blood sampling, were therefore included in the linear regression analyses. Frequencies of CM, RP, and metritis occurring up until the time of blood sampling (5 to 8 DIM) by treatment group were calculated using the frequency procedure (PROC FREQ). Values of NEFA and WBC counts were evaluated for normality and, where relevant, log10-transformed for inclusion in the statistical analyses.

Treatment effects on WBC counts were evaluated with mixed linear regression models (PROC MIXED). Fixed effects in the model included, as class variables, treatment (Control/PEG), parity (primiparous/multiparous), BCS (under: < 3; proper: 3 to 3.5 and over: > 3.5; [11]) and calving month (February/March, April, May, June/July); Pre-NEFA concentrations were log10-tranformed and included as a continuous variable. Two-way interactions with treatment and parity were checked for significance. Farm, as a class variable, was included as a random effect.

The initial statistical model looked like:

WBC count = intercept + treatment + parity + BCS + calving month + Log10(Pre-NEFA) + two-way interactions + farm(random) + error

In a first model (Model 1) we considered only pre-treatment variables included in the modeling process (treatment, parity, BCS, calving month, Pre-NEFA, farm (random)). This model evaluates the full treatment effect of PEG, including the intermediate effect that treatment may have on reducing the incidence of clinical disease in early lactation.

In a second model (Model 2) the effect of disease occurrence on WBC count is addressed separately by introducing disease occurrence in the model. In this model, the treatment effect is now separated into a direct treatment effect and an indirect effect through disease occurrence [20]. Clinical diseases (CM, RP or metritis) up until the time of blood sampling (5 to 8 DIM) and their two-way interactions with treatment were checked for significance in this second model.

After the initial full model lay-out, a backward stepwise selection process was performed. Given the design and objectives of this study, treatment and parity were always forced into the models. In the modeling process, only variables or interactions with a *P* < 0.10 remained in the model. Statistical tendency and statistical significance were decided at a *P* < 0.10 and a *P* < 0.05, respectively. The final model fit was evaluated using akaike’s information criterion and the normality of the distribution of the final model residuals.

## Results

### Study population

A total of 593 animals was sampled of which 6 animals did not meet the per protocol inclusion rule: 5 animals in the PEG group were injected only once and 1 cow in the control group was injected erroneously. Ten animals (Control = 6 and PEG = 4) were excluded because of too early enrollment (≥ 47 and up to 113 days before calving), 7 cows (Control = 6 and PEG = 1) because of an excessive length of previous lactation (≥ 600 days in milk) and 1 PEG cow because of a very low daily milk production in the previous lactation (1.6 L/day). A total of 26 animals had no Pre-NEFA determination (Control = 12, PEG = 14) and 2 Control cows had no WBC determination. Thus, 541 cows remained in the final analysis, representing the study population. The included cows according to parity and treatment were: 176 primiparous cows: Control = 87, PEG = 89 and 365 multiparous cows: Control = 181, PEG = 184.

In the study population, no differences (*P* > 0.4) where detected between treatment groups or between parity groups for the interval between enrollment and calving: mean and SD were 8 ± 5 days. Approximately 50% of the cows were enrolled within one week before calving, 43% within two weeks before calving, 5% within three weeks before calving and 2% more than three weeks before calving. Thus, 98% of the cows were enrolled within 21 days before calving.

Descriptive data of the previous lactation for the multiparous cows in the study population by treatment group are shown in Table 1. These descriptive data include parity, days open, previous milk production (kg/lactation), days in milk at dry off, daily milk production, somatic cell counts (log_10_-transformed) at the last test day of the previous lactation, and occurrence of one or more clinical mastitis cases. No significant differences between treatment groups were found in any of these variables.

**Table 1:** Descriptive data from the previous lactation for multiparous cows.

	Treatment (Mean ± SD)		
Item	Control	PEG	P-value
Lactation number at enrollment	2.1 ± 1.3	2.3 ± 1.3	0.19
Days open previous lactation	149 ± 102	143 ± 102	0.57
Previous lactation milk production*	7326 ± 2001	7374 ± 2202	0.83
Days in milk at dry-off	356 ± 87	347 ± 86	0.34
Daily milk production previous lactation**	21 ± 5	21 ± 5	0.36
Log_10_ SCC at dry-off	2.4 ± 2.3	2.4 ± 2.3	0.95
Occurrence of CM cases (%, n)***	41 (71/174)	40 (70/173)	0.95

* kg/lactation

** kg/day

***7 and 11 Control and PEG cows respectively have no previous data of CM.

Control = 181; PEG = 184.

No difference between treatment groups was found for BCS at enrollment (Control = 3.3 ± 0.4, PEG = 3.4 ± 0.4, *P* = 0.17). However, BCS was related to parity at enrollment (primiparous = 3.6 ± 0.3, multiparous = 3.3 ± 0.4, *P* < 0.01). After categorization by prepartum BCS, the number of animals by treatment group were: under-conditioned: Control = 34, PEG = 28, proper-conditioned: Control = 162, PEG = 172 and over-conditioned: Control = 72, PEG = 73.

No difference between treatment groups was found for Pre-NEFA concentration (Control = 0.5 ± 0.4, PEG = 0.5 ± 0.4, *P* = 0.22), but primiparous cows had higher Pre-NEFA concentrations than multiparous cows (0.6 ± 0.5 vs 0.5 ± 0.4 m*M*, *P* < 0.01).

In early lactation, up until the time of blood sampling, clinical disease occurrence was: CM: Control = 16, PEG = 15; RP: Control = 17, PEG = 20; metritis: Control = 42, PEG = 53.

### Effect of pegbovigrastim on white blood cell counts at 6 ± 1 days in milk in primiparous and multiparous cows

Overall, differences in least squares means (Tukey-Kramer adjustment) showed that treatment with PEG increased WBC count (Control = 12.2 ± 0.9, PEG = 21.7 ± 0.9 x10^3^/μL; *P* < 0.001), neutrophil count (Control = 5.7 ± 0.6, PEG = 13.6 ± 0.6 x10^3^/μL; *P* < 0.001), lymphocyte count (Control = 5.5 ± 0.3, PEG = 6.8 ± 0.3x10^3^/μL; *P* < 0.001) and monocyte count (Control = 0.41 ± 0.05, PEG = 0.68 ± 0.05x10^3^/μL; *P* < 0.001) at 6 ± 1 (mean ± SD) days in milk.

Table 2 shows the solutions for the final regression models. No parity effects were detected on WBC, neutrophil or monocyte counts. Primiparous cows tended to show lower lymphocyte counts (*P* = 0.08, Model 1), but this effect did not remain significant when early lactation disease was included in the analyses (Model 2). No BCS effects were detected for any of the cell types and there was no interaction of BCS with treatment.

**Table 2:** Solutions for the final regression models for white blood cell, neutrophil, lymphocyte and monocyte counts.

		Model 1			Model 2		
Cell type	Explanatory variable	Estimate	SE	*P*	Estimate	SE	*P*
WBC	Intercept	8.8	1.3	0.007	8.4	1.2	0.006
	Treatment	14.2	1.4	<0.001	15.6	1.4	<0.001
	Parity	-0.9	0.7	0.21	-0.8	0.7	0.24
	Pre-NEFA	-3.4	1.5	0.02	-4.0	1.4	0.005
	Calving month			0.02			0.01
	Pre-NEFA x Trt	3.4	1.8	0.07	3.4	1.8	0.06
	Calving month x Trt			<0.001			<0.001
	Metritis				-0.2	1.3	0.89
	Metritis x Trt				-4.8	1.8	0.008
Neutrophils	Intercept	3.4	0.9	0.03	3.1	0.8	0.03
	Treatment	11.8	1.0	<0.001	12.8	1.0	<0.001
	Parity	-0.1	0.5	0.84	-0.09	0.5	0.86
	Pre-NEFA	-1.8	1.0	0.08	-2.3	0.9	0.02
	Calving month			<0.001			<0.001
	Pre-NEFA x Trt	3.0	1.3	0.03	3.0	1.3	0.02
	Calving month x Trt			<0.001			<0.001
	RP				-0.7	1.4	0.96
	Metritis				-0.05	1.0	0.96
	RP x Trt				-3.9	2.0	0.04
	Metritis x Trt				-2.7	1.3	0.04
Lymphocyte	Intercept	4.7	0.5	0.002	4.6	0.5	0.002
	Treatment	1.3	0.4	<0.001	1.6	0.4	<0.001
	Parity	-0.7	0.4	0.08	-0.6	0.4	0.11
	Pre-NEFA	-2.0	0.5	<0.001	-2.0	0.5	<0.001
	Calving month			<0.001			<0.001
	Metritis				0.2	0.7	0.74
	Metritis x Trt				-2.0	1.0	0.03
Monocytes	Intercept	0.24	0.07	0.005	0.20	0.07	0.07
	Treatment	0.5	0.08	<0.001	0.40	0.08	<0.001
	Parity	-0.004	0.04	0.94	0.001	0.04	0.98
	Pre-NEFA	-0.12	0.08	0.16	-0.20	0.08	0.08
	Calving month			0.009			0.005
	Pre-NEFA x Trt	0.3	0.1	0.02	0.2	0.1	0.02
	Calving month x Trt			0.04			0.04
	Metritis				-0.04	0.08	0.63
	CM				0.2	0.1	0.04
	Metritis x Trt				-0.2	0.1	0.09

Primiparous: Control = 87, PEG = 89; multiparous: Control = 181, PEG = 184.

Model 2: Includes early lactation disease occurrence, up until the time of blood sampling: CM: Control = 16, PEG = 15; RP: Control = 17, PEG = 20; metritis: Control = 42, PEG = 53.

Reference groups: Month 3, Control group and multiparous cows. *White blood cells. Pre-NEFA: prepartum NEFA concentrations, CM: clinical mastitis, RP: retained placenta, Trt: treatment.

Model 1 includes treatment and parity, model 2 also includes the effect of clinical disease on white blood cell counts.

As Table 2 shows, there was an association of Pre-NEFA concentration with decreased WBC counts (*P* = 0.02 and *P* = 0.005, Model 1 and 2, respectively). Pre-NEFA concentration tended to be associated with decreased neutrophil counts (*P* = 0.08, Model 1), which became significant when disease occurrence was included in the model (*P* = 0.02, Model 2). Moreover, Pre-NEFA concentration was associated with decreased lymphocyte counts (*P* < 0.001, Model 1 and 2) and tended to be associated with decreased monocyte counts (*P* = 0.08, Model 2).

**Fig 2:**
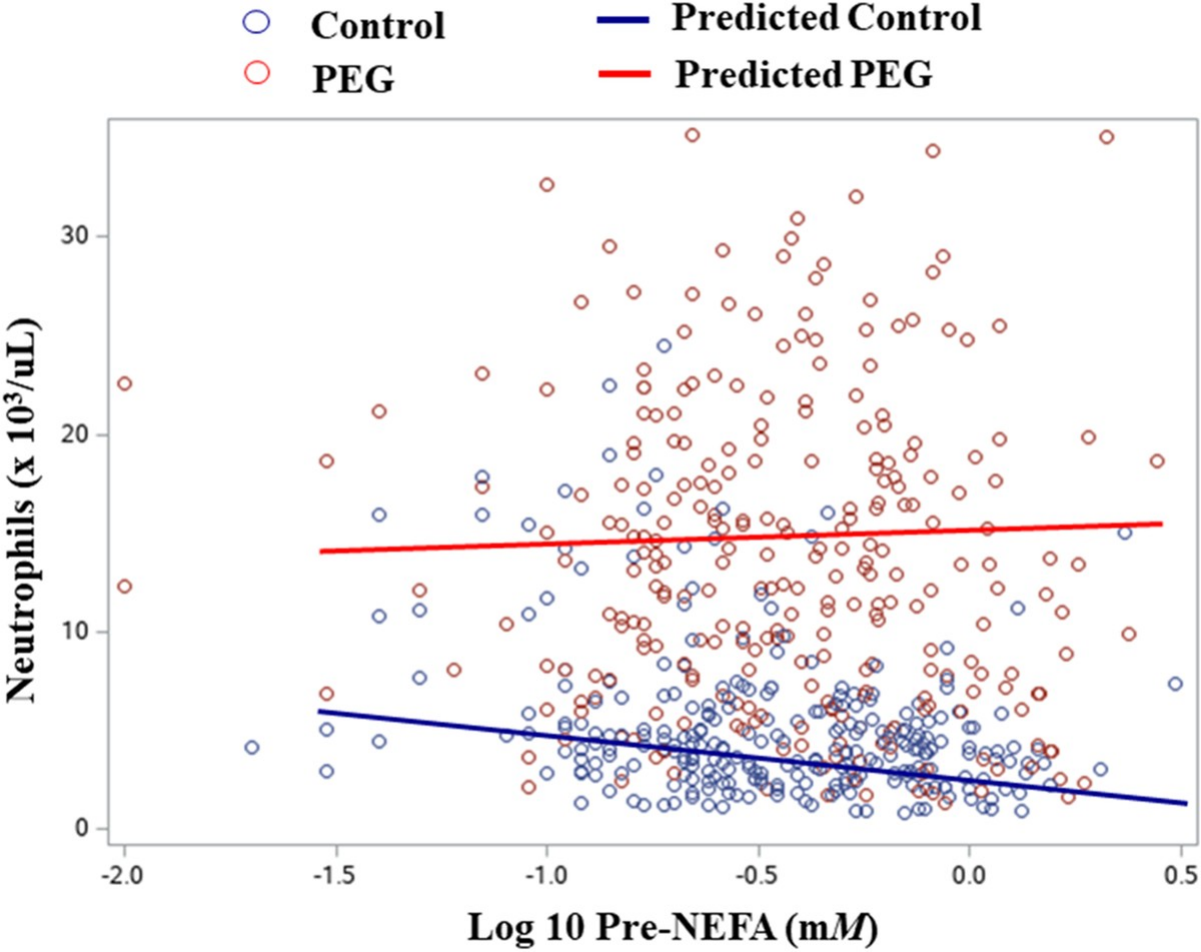
Observed values and prediction lines for neutrophil x 10^3^/μL by log10-transformed prepartum NEFA concentrations (m*M*). Control cows = 268; PEG cows = 273.

Prepartum NEFA concentration tended to interact with treatment for WBC counts (*P* = 0.07 and *P* = 0.06, Model 1 and 2, respectively), and significantly interacted with treatment for neutrophil (*P* = 0.03 and *P* = 0.02, Model 1 and 2, respectively) and monocyte counts (*P* = 0.02, Model 1 and 2). Fig 2 presents observed values and prediction lines (model 2) for neutrophil counts by log10-transformed Pre-NEFA concentrations in control and PEG cows.

Model 2, that included the modifying effect of disease on cell counts (Table 2), showed that CM occurrence was associated with increased monocyte counts (*P* = 0.04). A treatment by RP interaction was observed for neutrophil counts (*P* = 0.04); in PEG treated cows, RP occurrence was associated with decreased neutrophil counts compared to PEG treated cows without RP. Similarly, a treatment by metritis interaction was shown for WBC, neutrophil and lymphocyte counts (*P* = 0.008, *P* = 0.04 and *P* = 0.03, respectively) and a tendency for this interaction was shown for monocyte counts (*P* = 0.09). In PEG treated cows, metritis occurrence was associated with decreased WBC, neutrophil and lymphocyte counts and tended to be associated with decreased monocyte counts compared to PEG treated cows without metritis.

**Fig 3:**
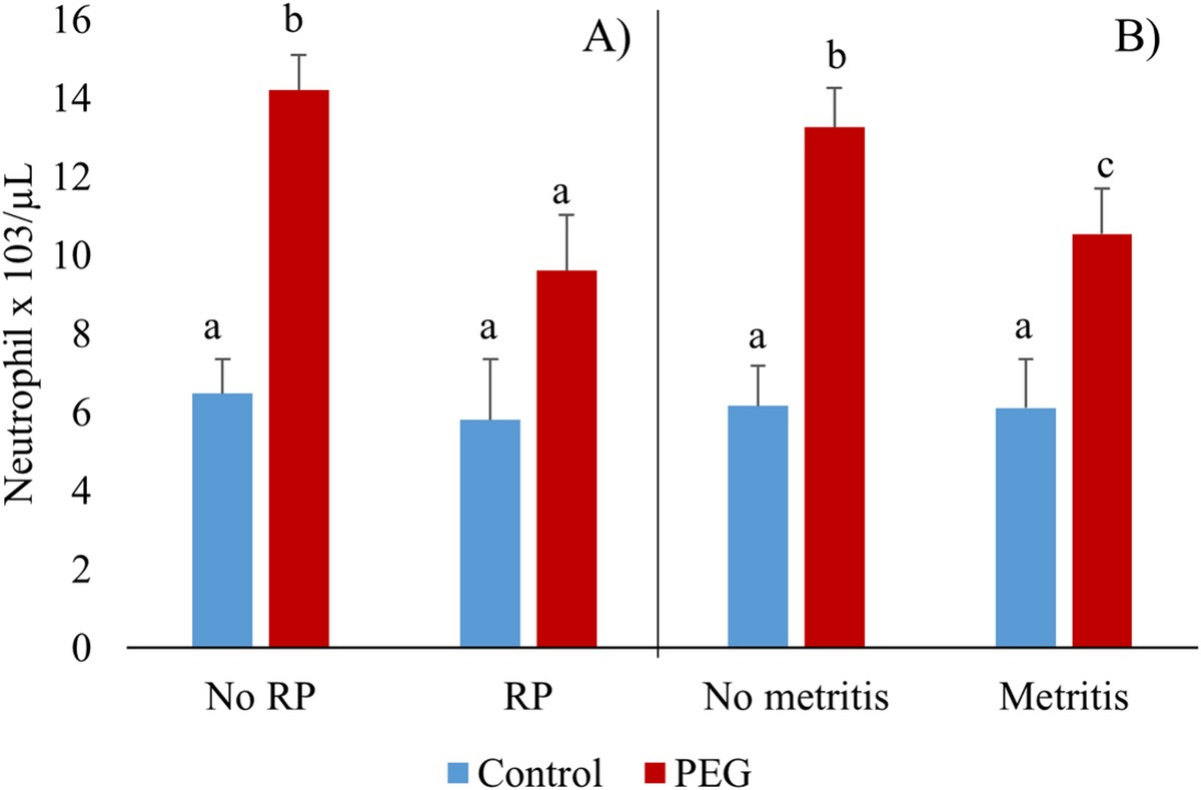
Blood neutrophil counts/μL at 6 ± 1 (mean ± SD) days in milk in cows diagnosed with: retained placenta (RP): Control = 17, PEG = 20, No RP: Control = 251, PEG = 253 (Panel A); metritis: Control = 42, PEG = 53, No metritis: Control = 226, PEG = 220 (Panel B). Different letters, *P* < 0.05.

Fig 3 shows least square means of neutrophil count differences (Tukey-Kramer adjustment) for RP (Panel A), and metritis (Panel B) in Control and PEG cows. In the PEG treated group, cows with RP and metritis showed lower neutrophil counts than PEG treated cows without these clinical diseases (*P* = 0.006 and *P* = 0.005, respectively).

Calving month showed an interaction with PEG treatment for WBC, neutrophil and monocyte count (Table 2).

## Discussion

In this randomized controlled trial on four commercial grazing dairy farms, we tested the hypothesis that parity affects the WBC counts in response to PEG. Additionally, the association of prepartum BCS and Pre-NEFA concentration with WBC counts in control and PEG cows was investigated. Our data confirmed that PEG treatment increased WBC, neutrophil, lymphocyte and monocyte counts in grazing dairy cows and that this effect was independent of parity.

In the Control group, an increased Pre-NEFA concentration was associated with reduced WBC, neutrophil and lymphocyte counts and tended to be associated with reduced monocyte counts. Treatment with PEG increased neutrophil and monocyte counts independent of Pre-NEFA concentration. The effect of treatment on WBC counts also tended to be independent of Pre-NEFA concentration, thereby reverting the negative association of Pre-NEFA concentration with neutrophil and monocyte counts and tending to revert the negative association of Pre-NEFA with WBC counts.

A limitation of this study is that WBC counts were not measured before treatment. Nevertheless, as this was a controlled clinical trial with a large number of animals and a robust randomization as shown by the balance between treatment groups, it is unlikely that significant pre-treatment differences in WBC counts could bias the study results.

The observation that treatment with PEG resulted in higher WBC, neutrophil, lymphocyte and monocyte counts at 6 ± 1 (mean ± SD) days in milk was expected [9, 23]. Although a lack of PEG effect on monocyte counts has previously been reported [31], our work is, on the whole, consistent with these previous studies [9, 23]. The magnitude of the increase in neutrophil counts (2.5-fold) is similar to previous reports on commercial farms [23, 32], but lower than reports from studies under more experimental conditions (6-fold change, [9]). This could be due to variations in age, diet, body condition, disease occurrence or other variables [25] that may play a role under commercial conditions. Besides, calving month in our study had an effect on all cell type counts and interacted with treatment, which may be related to the importance of environmental and management conditions in grazing systems [33]; e.g., cows in late gestation under overstocked conditions suffered stress as well as changes in energy metabolism compared to animals that were not overstocked [34].

The increase in WBC counts caused by PEG treatment was not affected by parity. Both groups were exposed to virtually the same environmental and management conditions. Our data is consistent with previous studies [24] that suggested that PEG improves migration, adhesion and antimicrobial capacity and enhances the inflammatory response regardless of parity. Neither was the increase in WBC counts by PEG treatment affected by BCS. In this study BCS was measured only at enrollment. In other reports, BCS loss during transition has been associated with an increased NEFA concentration [12, 35].

Disease occurrence affected only monocyte counts; CM occurrence was associated with increased counts. Interestingly, when disease occurrence was included in the model the negative association between Pre-NEFA concentrations with WBC, neutrophil and monocyte counts became more evident. When controlling for disease impact, Pre-NEFA concentrations showed a profound negative effect on early lactation WBC counts. The difference between the two models would indicate that animals showing clinical signs of disease would have both higher NEFA and WBC values. Correcting for disease occurrence in the statistical modeling will then make the negative relationship between Pre-NEFA and WBC more visible.

Overall, our data is consistent with previous reports on the association between increased NEFA concentrations and the decline in WBC counts [8]. Neutropenia has been reported during the first and second week in lactation [5, 22], a period of time that has also been associated with low blood glucose and increased NEFA concentrations in modern high-producing dairy cows [36]. Glucose is required by neutrophils for proliferation, survival and differentiation [4]. Moreover, it has been reported that NEFA concentrations decreased the viability of neutrophils and increased necrosis of these cells [10]. It has also been reported that blood neutrophils shortly after parturition are more prone to apoptosis [37].

This is the first study reporting the interaction of Pre-NEFA concentrations and PEG treatment with neutrophils and monocytes: PEG treatment reversed the decline of neutrophil and monocyte counts in early lactation associated with high Pre-NEFA concentrations. Model results as shown in Table 2 indicate that, with increasing Pre-NEFA concentrations, WBC counts decrease in control cows. However this negative relationship between Pre-NEFA and WBC counts was not observed in PEG treated cows and there was even a slight increase in neutrophil counts. It was previously [9] reported that the treatment effect of PEG was not affected by prepartum energy restriction, which is in accordance with our results. However, McDougall et al. [9] did not observe a negative effect of Pre-NEFA on WBC counts in untreated control cows, which contrasts with our observations. In their trial [9], a high percentage of cows had high Pre-NEFA concentrations in both the feed-restricted (85%) and the control group (56%), thereby making it more difficult to evaluate the relationship between high Pre-NEFA and PEG treatment.

Pegbovigrastim sharply increased the blood neutrophil counts in 24 hours due to the release from the bone marrow pools [31], and reportedly also increased the expression of genes related with cell survival [38]. Taking into account these results, we hypothesize that PEG treatment in cows with more pronounced NEB and decreased WBC counts would cause a restoration of the WBC count to levels observed in cows with better energy balance and thus, PEG treatment would have a stronger preventive effect against disease in high Pre-NEFA cows than in cows with normal values. Further studies relating the effect of PEG treatment with disease occurrence during transition and its association with metabolism are warranted.

Pegbovigrastim reversed the negative association of Pre-NEFA concentration with neutrophil counts and monocyte counts, and, likely as a consequence, tended to prevent the negative association of Pre-NEFA with WBC counts. However, this was not observed for lymphocyte counts. Among other differences, neutrophils and monocytes are both derived from myeloblasts while lymphocytes derive from lymphoid progenitors [31]. It may be hypothesized that PEG could have different immune restoration mechanisms according to the cell type.

In PEG treated cows, an association of neutrophil count with clinical disease was detected. In the PEG group, animals diagnosed with RP or metritis showed lower neutrophil counts compared to treated cows without these clinical diseases, while this reduction was not observed in PEG treated CM cows. However, Zinicola et al. [23] reported that PEG treated cows diagnosed with both CM and metritis had lower WBC counts than PEG treated cows without the clinical event. These authors also reported that PEG treated metritis cows had higher neutrophil counts in the vagina than control cows with metritis. All these observations are consistent with the hypothesis of Ruiz et al. [28] that PEG elicits a more robust (or longer lasting) intra-uterine migration of neutrophils.

## Conclusions

Our data confirm that PEG treatment increases WBC, neutrophil, lymphocyte and monocyte counts in grazing dairy cows and that this effect is independent of parity. In control cows, Pre-NEFA concentration was associated with reduced WBC, neutrophil and lymphocyte counts and tended to be associated with reduced monocyte counts. In this study it was shown that PEG treatment reverted the negative association of Pre-NEFA concentration with neutrophil counts and monocyte counts, and tended to revert the negative association of Pre-NEFA concentration with total WBC count.
